# Inter- and intraspecific phenotypic plasticity of three phytoplankton species in response to ocean acidification

**DOI:** 10.1098/rsbl.2016.0774

**Published:** 2017-02

**Authors:** Giannina S. I. Hattich, Luisa Listmann, Julia Raab, Dorthe Ozod-Seradj, Thorsten B. H. Reusch, Birte Matthiessen

**Affiliations:** GEOMAR, Helmholtz Centre for Ocean Research Kiel, Düsternbrooker Weg 20, 24105 Kiel, Germany

**Keywords:** *Emiliania huxleyi*, *Gephyrocapsa oceanica*, *Chaetoceros affinis*, ocean acidification, plasticity, reaction norm

## Abstract

Phenotypic plasticity describes the phenotypic adjustment of the same genotype to different environmental conditions and is best described by a reaction norm. We focus on the effect of ocean acidification on inter- and intraspecific reaction norms of three globally important phytoplankton species (*Emiliania huxleyi, Gephyrocapsa oceanica* and *Chaetoceros affinis*). Despite significant differences in growth rates between the species, they all showed a high potential for phenotypic buffering (similar growth rates between ambient and high CO_2_ conditions). Only three coccolithophore genotypes showed a reduced growth in high CO_2_. Diverging responses to high CO_2_ of single coccolithophore genotypes compared with the respective mean species responses, however, raise the question of whether an extrapolation to the population level is possible from single-genotype experiments. We therefore compared the mean response of all tested genotypes with a total species response comprising the same genotypes, which was not significantly different in the coccolithophores. Assessing species reaction norms to different environmental conditions on short time scale in a genotype-mix could thus reduce sampling effort while increasing predictive power.

## 1. Introduction

The expression of different phenotypes of a genotype in different environments is called phenotypic plasticity. It is described by the shape of the reaction norm of a trait value at different environments. No visible change in a focal trait despite a change in the environment (horizontal reaction norm) is defined as phenotypic buffering [1]. This does not preclude changes in other traits or on the molecular level. How phenotypic plasticity interacts with evolutionary adaptation is contentious [2]; it is discussed to be both a non-mutual alternative to evolutionary adaptation and a strong driver for adaptation. In the plasticity-first scenario, a population/species survives environmental change due to pronounced plasticity until genetic mutations may occur and potentially fix the previously plastic trait such that the fitness under the new conditions increases [3]. Provided that there is standing genetic/genotypic variation, mean population fitness can also increase at the level of populations, resulting from alteration of gene/genotypic frequencies over time caused by selection.

One prominent environmental change is ocean acidification (OA) [4], describing changes in the carbonate system due to anthropogenic CO_2_ dissolving in the ocean, which potentially affects organisms, species and communities [5]. In marine phytoplankton, different effect sizes and signs in response to OA (i.e. varying reaction norms) have been observed between and within different taxa [6]. A reason for within species differences can be adaptation to different geographical regions [7]. Little is known, however, about inter- and intraspecific variation in reaction norms of populations and communities originating from one geographical region. Additionally, Valladares *et al*. [8] summarize that current mathematical models predicting alterations in communities due to climate change lack data on intraspecific genetic and phenotypic variation. Largely diverging responses to OA of different *Emiliania huxleyi* genotypes among studies [9] raise the question of whether responses derived from one or a few genotypes can be directly extrapolated to the population and community level.

We compare (i) the intra- and interspecific reaction norms of three phytoplankton species in response to two different CO_2_ conditions and (ii) the total multi-genotype species response to the mean intraspecific CO_2_-response of the respective species. The species used include two common bloom forming coccolithophores, *E. huxleyi* and *Gephyrocapsa oceanica,* and a diatom, *Chaetoceros affinis*, originating from one region*.* We expect that (i) the coccolithophores show a zero to negative reaction norm as a result of OA [10] compared with a positive slope for the diatom as a result of profitable dissolved inorganic carbon use [11]. Furthermore, (ii) genotypes of a species should differ in their growth response, and (iii) the total species reaction norm is unequal to that of single genotypes but similar to the calculated mean reaction norm of all genotypes together.

## 2. Material and methods

From *C. affinis*, *E. huxleyi* and *G. oceanica* nine different genotypes each and one mix of all genotypes with equal initial abundances (electronic supplementary material, S1) were immediately (i.e. without acclimation) exposed to ambient and high CO_2_ concentration in order to obtain a two-point reaction norm within the acclimation phase. All cultures used were field isolates (2014–2015) originating from one geographical region (Gran Canaria, 27°59′ N 15°22′ W). This design allowed us to compare the within and among species plasticity of one community and the effect of intraspecific interaction on the short-term CO_2_-response by contrasting the multi-genotype total species (mixculture) to the mean intraspecific plastic (monoculture) response. All treatment combinations were threefold replicated resulting in 180 experimental units (0.5 l polycarbonate bottle). Owing to space limitation each species was tested separately (June to July 2016; electronic supplementary material, figure S1).

The ambient and high CO_2_-treatment was manipulated by aerating the artificial-seawater (35 salinity; after [12]) for 24 h with CO_2_-enriched air (400 and 1250 ppm, respectively) prior to the experiment. The dissolved inorganic carbon [13] was 2164.68 ± 27.76 and 2307.94 ± 51.59 µmol kg^−1^ with a total alkalinity (following [14]) of 2442.04 ± 20.72 and 2456.30 ± 20.63 µmol kg^−1^ for ambient and high CO_2_, respectively. Nutrients were added to the final concentrations of 19.98 ± 0.39 µmol l^−1^ nitrate, 1.01 ± 0.07 µmol l^−1^ phosphate and 4.40 ± 0.24 µmol l^−1^ silicate for coccolithophores and 34.16 ± 0.30 µmol l^−1^ silicate for the diatoms. The excess of silicate added to medium used for diatoms ensured that all species were limited by nitrate in the experiment, and was a prerequisite to compare results among species. Vitamin and trace metals were added in *f*/8 concentration [15]. The prepared medium was sterile filtered (0.2 µm pore size) into the experimental units. Each experimental unit was inoculated with an initial total biovolume of 8280 µm per millilitre of exponentially growing cells, balancing the substantial differences in cell size of the species used.

The experiment was carried out under constant rotation (0.75 min^−1^) at 20°C and a 17 L : 7 D cycle reaching a maximum light intensity of 350 µmol m^−2^ s^−1^ 3 h after dusk and dawn. The development of each culture was followed by daily cell counts for the coccolithophores (Z2TM COULTER COUNTER^®^) and fluorescence measurements for the diatom (10AU Field and Laboratory Fluorometer by Turner Designs). The total sampling volume was below 10%. Cultures were terminated at the third day in stationary phase (experimental duration: 9–16 days).

For statistical analysis, the software R was used [16]. Growth rates were determined for each replicate by fitting an exponential growth model inbuilt in the package ‘growthrates’ [17]. The overall effect of CO_2_, species and genotype on growth rate was tested using a nested ANOVA (growthrate∼ CO_2_ * Species * (Genotype/Species)). Subsequent analysis of intraspecific plasticity and the effect of genotype was tested by separate ANOVAs for each species (growthrate ∼ CO_2_ * Genotype) and genotype (growthrate∼CO_2_) and visualized as the difference in growth rates between ambient and high CO_2_ [18]. The difference between mean interspecific plastic effects and the multi-clonal total species response was tested for each species separately (growthrate∼Mono-/Mix-culture). Parametric assumptions were explored graphically.

## 3. Results

**Figure 1.**
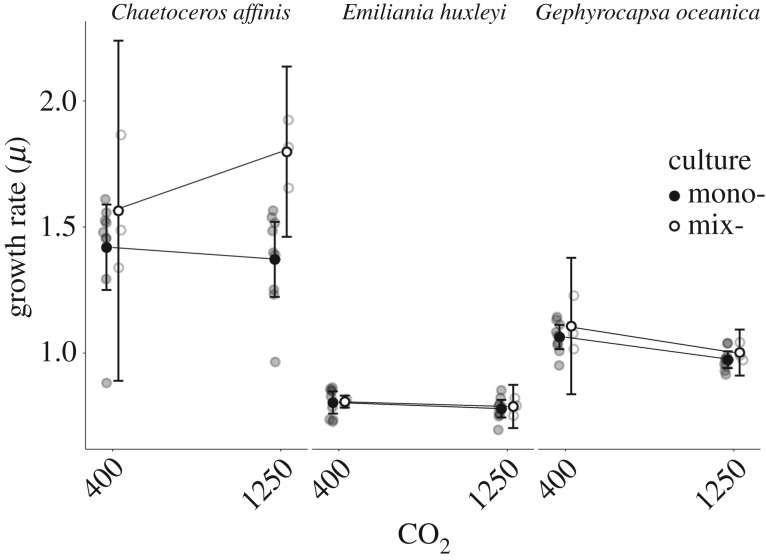
Two-point reaction norm of growth rates in ambient and high CO_2_ across mean of each genotype grown in monoculture (closed circle, *N* = 9 (nine genotypes)) and a mixculture of all genotypes (open circle, *N* = 3 (three replicates)) for each species. Mean and 95% CI are shown.

**Figure 2.**
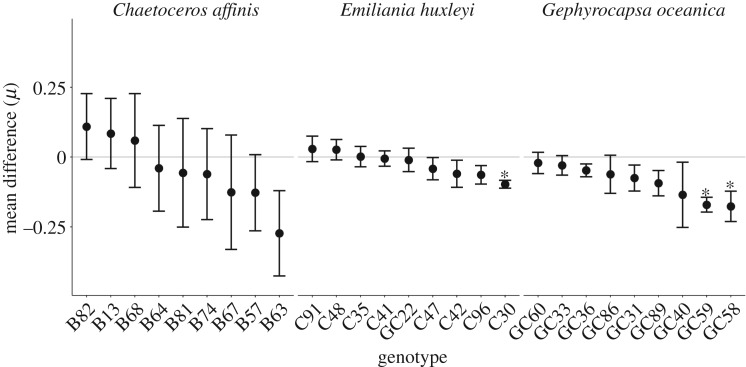
Mean difference and its standard error of growth rates (*μ*) between high and ambient CO_2_ of each genotype and species. Grey line indicates no difference in growth between CO_2_-treatments and asterisks highlight genotypes where growth rate was significantly affected by CO_2_.

The growth rates (*μ*) of the different species were significantly different (*F*_2,132_ = 355.586, *p* < 0.001), with *E. huxleyi* and *G. oceanica* showing a 44% and 28% lower *μ* than *C. affinis* (figure 1). Across all species *μ* was generally lower in high CO_2_ and significantly depended on genotype (*F*_1,132_ = 8.433, *p* = 0.004; *F*_24,132_ = 6.161, *p* < 0.001; respectively). Analysis on the species level revealed that only the *μ* of *G. oceanica* was significantly lower in high CO_2_ (*F*_1,56_ = 20.659, *p* < 0.001). Furthermore, the magnitude of the difference in *μ* between the CO_2_-treatments was not uniform among all tested genotypes within each species (figure 2). While the mean difference of *C. affinis* genotypes ranged from 0.109 to −0.273 with a substantial standard error, those of *E. huxleyi* had a narrow range from 0.029 to −0.097, with one genotype (C30) showing a significantly lower *μ* under high than under ambient CO_2_ (*F*_1,4_ = 48.64, *p* = 0.002). The general negative mean difference in *G. oceanica* genotypes ranged from −0.17 to −0.21. Two genotypes (GC59 and GC58) were significantly negatively affected by CO_2_ (*F*_1,4_ = 10.7, *p* = 0.031; *F*_1,4_ = 42.12, *p* = 0.003), which drove the overall significant negative effect of CO_2_ on the *μ* of *G. oceanica*. Finally, the difference in *μ* between mono- and mixcultures was significantly different only in *C. affinis* (*F*_1,22_ = 8.405, *p* = 0.008) with a higher *μ* in the mix- than in the monocultures.

## 4. Discussion

We assessed the variation of phytoplankton acclimation reaction norms in two potential ‘loser’ and one potential ‘winner’ species under OA. Interestingly, all three species mostly buffered the effect of CO_2_ and thus showed a mean reaction norm slope similar or close to zero. However, within species, the response range varied. *C. affinis* showed the largest range in growth rates among genotypes tested. *E. huxleyi* and *G. oceanica* are ecologically more alike each other than *C. affinis* which could explain a more similar negative response among them. Owing to the extensive literature [19] showing negative effects of OA on coccolithophores, we expected to see more genotypes showing a significant negative effect in growth under high CO_2_, but note that most of those measurements were taken after acclimation, while our study was designed to address exactly the acclimation phase. Nevertheless, in line with the literature, we found that *G. oceanica* was most and significantly negatively affected by high CO_2_ [10]. Overall the weak effect of CO_2_ could partly be due to the high variability among replicates masking a potential difference in growth rate between the two treatments. Additionally the experimental level of CO_2_ in this study may be within the natural range (daily fluctuations, upwelling) species experience and can be phenotypically buffered [20].

The effect of CO_2_ on single genotypes differed compared with the mean species response. *E. huxleyi*, for example, showed no overall effect of CO_2_ on growth even though one genotype grew significantly slower under high CO_2_. We observed the opposite in *G. oceanica*, with an overall negative effect of high CO_2_ on growth rate even though seven out of nine genotypes showed no difference. Our findings highlight the importance of testing many genotypes rather than using single genotypes, as has been done in most studies so far [19], to avoid over- or underestimation of a species reaction norm to climate change.

The question remains how to minimize the sampling effort needed to study reaction norms of a representative set of genotypes of a species. We here show that the reaction norm of a culture containing the full set of genotypes compared with the mean of all genotypes cultured singly was similar in both coccolithophores but not in the diatom. The significant effect of culture condition on the slope in *C. affinis* could be driven by the high variability within the three replicates in the mixcultures. Nevertheless, our results suggest that the use of a mixculture of genotypes is sufficient to assess a species reaction norm on short time scales. This largely suggests that the total species reaction norm obtained from the mixcultures reflects the mean species plasticity if, as assumed here, genotype loss due to sorting is likely to be negligible (electronic supplementary material, S2).

Our experiments highlight the importance of investigating species reaction norms rather than reaction norms of single genotypes to better predict reactions to short-term environmental change. We suggest that analysing a mix of genotypes is potentially an achievable and feasible way to identify more realistic species reaction norms. Nevertheless, future studies should assess final genotype sorting to fully understand the species reaction norms.

## Supplementary Material

Supplementary Hattich et al.

## Data accessibility

PANGEA https://doi.pangaea.de/10.1594/PANGAEA.865212 [21].
