# Supplementary material for: Inter- and intraspecific phenotypic plasticity of three phytoplankton species in response to ocean acidification
Source: Biol Lett. 2017 Feb;13(2):20160774. doi: 10.1098/rsbl.2016.0774 (PMC5326507; doi:10.1098/rsbl.2016.0774)
Supplement: Supplementary Hattich et al. [file rsbl20160774supp1.pdf]

Supplementary

S1: Genotype isolation and genotyping

All genotypes used were isolated from the same geographical region in the North Atlantic (south-east off Gran Canary, Spain, 27°59’N 15°22’W) between spring 2014- spring 2015. All genotypes of one species, with one exception, were isolated at once from a 2L seawater sample. More specifically, the *C affinis* genotypes were isolated in December 2014, while *G. oceanica* was isolated in February 2015 and *E. huxleyi* were collected and isolated in February 2014 (except for GC22 which was isolated in February 2015). All genotypes used in this experiment will be deposited in the Roscoff Culture Collection (http://roscoff-culture-collection.org/; Table S1)

All coccolithophore strains (i.e. *E. huxleyi* and *G. oceanica*) were genotyped using microsatellite (msat) analysis to assure that each culture used is a different strain (genotype). For *C. affinis* the msats are still under development. We nevertheless assume that all isolated single strains belong to a different genotype. Firstly, isolation of genotypes of all species followed the same protocol. Secondly, literature has shown that isolation of the same genotype from an amount of water, like we sampled in this study, is unlikely [1,2].

For microsatellite analysis DNA was extracted by adding 10 µL of TE-buffer to resuspend pellet of coccolithophoers. Samples were then sonificated for 3 minutes at 100% and then incubated for 1h at 56°C. Microsatellite amplification was done in the following reaction mix: 2.5µL multiplex mastermix (Qiagen), 0.25 µL 5pM forward and reverse primer (primers EHMS15b and P02E10b for *E. huxleyi* [3], primers GE06 and GE07 (Table S2.) for *G. oceanica*), 1 µL Q solution (Qiagen), 0.5 µL H2O and 0.5 µL of DNA template. The PCR reaction run for msat amplification was set up as follows: an initial phase of 15min at 95°C, 30 cycles of 30sec at 94°C, 90sec at 57°C and 1min at 72°C and final step of 30 min at 60°C. 1 µL PCR products was then added to a mix of ROX and Hidi (Qiagen) of 0.25 µL and 8.75 µL respectively and incubated for 3min at 94°C to denature double stranded products. The sequencer 3130xl Genetic Analyzer (Applied Biosciences) was then used to analyse microsatellite composition of each sample and data was analysed using GeneMarker software.

Table S1: All genotypes used are listed in the column “manuscript” under the name used in here and will be deposited in the Roscoff Culture Collection (RCC) under the name shown in the “RCC” column.

C. affinis		*E. huxleyi*		*G. oceanica*	
**Manuscript**	**RCC**	**Manuscript**	**RCC**	**Manuscript**	**RCC**
**B13**	EHGLL13B	**C48**	EHGKLC48_20	**GC31**	EHGLLGC31
**B75**	EHGLL57B	**C30**	EHGKLC30_20	**GC33**	EHGLLGC33
**B63**	EHGLL63B	**C35**	EHGKLC35_20	**GC36**	EHGLLGC36
**B64**	EHGLL64B	**C91**	EHGKLC91_20	**GC40**	EHGLLGC40
**B67**	EHGLL67B	**C96**	EHGKLC96_20	**GC58**	EHGLLGC58
**B68**	EHGLL68B	**C47**	EHGKLC47_20	**GC59**	EHGLLGC59
**B74**	EHGLL74B	**C41**	EHGKLC41_20	**GC60**	EHGLLGC60
**B81**	EHGLL81B	**C42**	EHGKLC42_20	**GC86**	EHGLLGC86
**B82**	EHGLL82B	**GC22**	EHGLLGC22	**GC89**	EHGLLGC89

Table S2: Primers used for *G. oceanica* msat analysis (strains N=9). Transcriptome as basis stems from Marine Microbial Eukaryote Transcriptome Sequencing Project (MMETSP) [4]**and is called Gephyrocapsa-oceanica-RCC1303**

Primer name	Sequence	Product length (from test)	Msat repeat unit	No. Alleles
**GE06Fm**	GTAATTGTCGTACGCCCG	162	$(CT)_{17.5}$	8
**GE06Rm**	CCAGGAATAGACTTAGGCCG			
**GE07Fm**	TGTCTCAGAGTCTCGCGG	189	$(TC)_{19}$	8
**GE07Rm**	GAGTTTGTGGCTGTCCTTTC			

S2: Relative contribution of genotypes in mixcultures

The mixcultures consisted of all genotypes of one species that initially contributed the same number of cells/mL. The relative contribution of each genotype was therefore 11 % at the start of the experiment (Figure S1). To avoid differences in initial concentrations of the respective genotypes', the mixcultures were inoculated from a stock mixculture prepared just before the start of the experiment. As in the monoculture each experimental unit was inoculated with an initial total biovolume of 8280 μm_3*ml^{-1} cells, resulting in an initial concentration of 20 cells*ml^{-1}, 180 cells*ml^{-1} and 24 cells*ml^{-1} for *C. affinis*, *E.huxleyi* and *G. oceanica*, respectively.

Change in the relative contribution of genotypes in the mixcultures was not tested over the course of the experiment. While we assumed that the relative genotypic contribution changes over time [5], we, however, did not expect an actual loss of genotypes in this experiment with only one batch cycle. Personal experience with the same *E. huxleyi* genotypes showed that a significant change in relative abundance of the genotypes occurs after an experimental duration of four batch cycles while only one genotype was actually lost after this time. This suggests that genotypic exclusion in *E. huxleyi* takes longer than the experimental duration of this study. It also concurs with Lohbeck et al. (2012) demonstrating that after 160 days (corresponding to ~ 30 batch cycles) only half of the initially present six *E. huxleyi* genotypes were lost [6]. The maintenance of genotypes in comparatively short-termed experiments can, however, also depend on the number of initially present genotypes. Sjöqvist and Kremp (2016) showed that all genotypes in *Skeletonema marinoi* mixcultures remained present if the cultures started with lower genotypic richness (i.e. 5 genotypes). In contrast higher initial genotypic richness (i.e. 20 genotypes) enhanced exclusion [7]. The use of nine genotypes in this study corresponds to a midrange genotype richness compared to Sjöqvist and Kremp´s study,and thus supports that genotype exclusion is expected to be low or almost negligible in our short term experiment.

However in future studies the negligible sorting and loss of genotypes over a short period of time (as argued above) should be assessed by analysis of the final genotype contribution after the experiment. This way the species reaction norm can be fully understood.

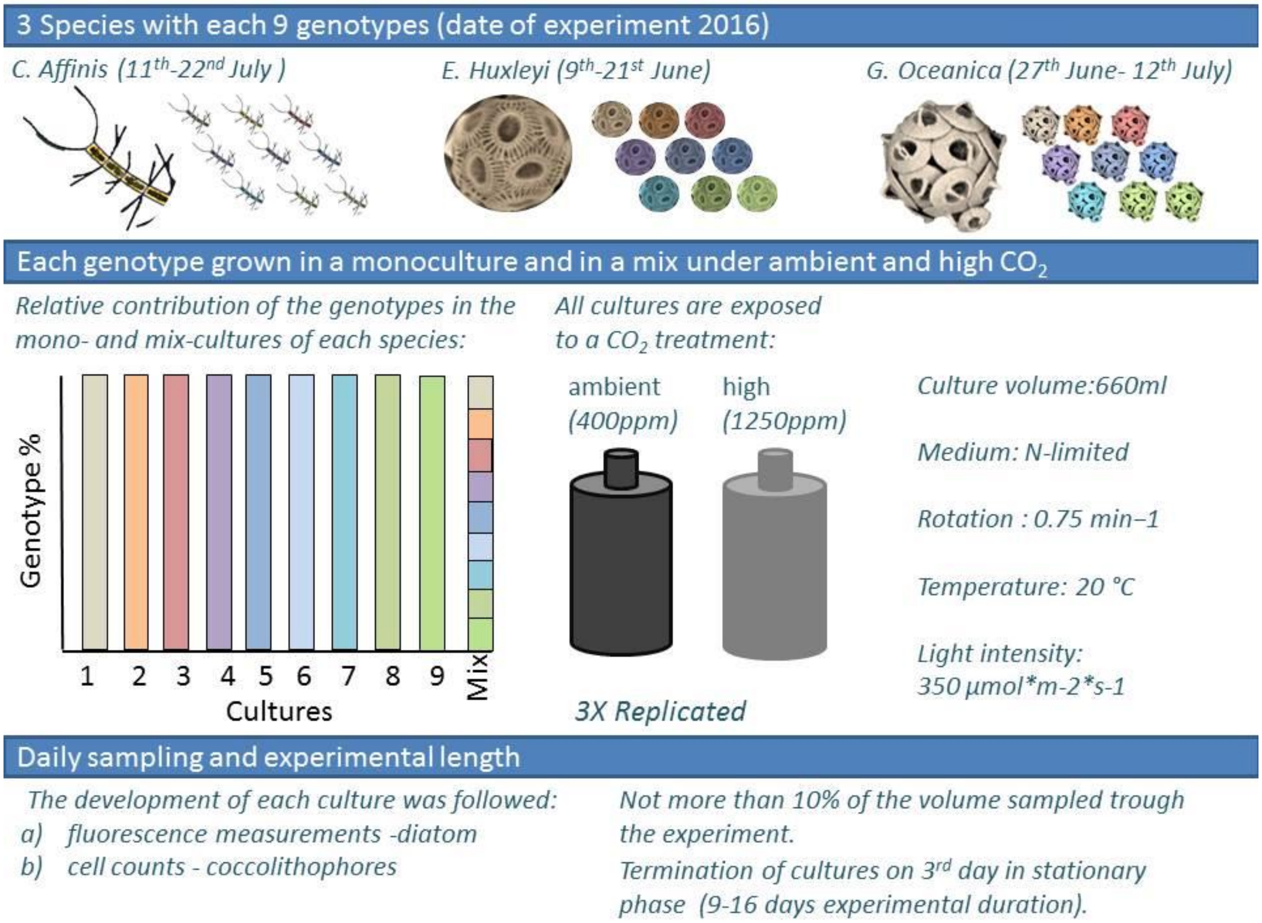

Figure S1: Overview of the experimental set up. Species and genotypes at the top, followed by general information of the cultivation as well as the CO_2 treatments and genotype composition in Mono- and Mixcultures. In the initial phase of the mixculture each of the nine genotypes contributes equally and is present with 11%. At the bottom are information about daily sampling as well as experimental length.

References

1. Evans, K. M., Bates, S. S., Medlin, L. K. & Hayes, P. K. 2004. Microsatellite marker development and genetic variation in the toxic marine diatom Pseudo-Nitzschia Multiseries (Bacillariophyceae). (doi:10.1111/j.1529-8817.2004.04027.x)

2. Rynearson, T. A. & Armbrust, E. V. 2005 Maintenance of clonal diversity during a spring bloom of the centric diatom Ditylum brightwellii. *Mol. Ecol.* **14**, 1631–1640. (doi:10.1111/j.1365-294X.2005.02526.x)

3. Debora Iglesias-Rodriguez, M., Schofield, O. M., Batley, J., Medlin, L. K. & Hayes, P. K. 2006 Intraspecific genetic diversity in the marine coccolithophore Emiliania huxleyi (Prymnesiophyceae): The use of microsatellite analysis in marine phytoplankton population studies. *J. Phycol.* **42**, 526–536. (doi:10.1111/j.1529-8817.2006.00231.x)

4. Keeling, P. J. et al. 2014 The Marine Microbial Eukaryote Transcriptome Sequencing Project (MMETSP): Illuminating the Functional Diversity of Eukaryotic Life in the Oceans through Transcriptome Sequencing. *PLoS Biol.* **12**, e1001889. (doi:10.1371/journal.pbio.1001889)

5. Roger, F., Godhe, A. & Gamfeldt, L. 2012 Genetic Diversity and Ecosystem Functioning in the Face of Multiple Stressors. *PLoS One* **7**. (doi:10.1371/journal.pone.0045007)

6. Lohbeck, K. T., Riebesell, U. & Reusch, T. B. H. 2012 Adaptive evolution of a key phytoplankton species to ocean acidification. *Nat. Geosci.* **5**, 346–351. (doi:10.1038/ngeo1441)

7. Sjöqvist, C. O. & Kremp, A. 2016 Genetic diversity affects ecological performance and stress response of marine diatom populations. *ISME J.* , 1–12. (doi:10.1038/ismej.2016.44)
